# Enhancing date seed phenolic bioaccessibility in soft cheese through a dehydrated liposome delivery system and its effect on testosterone-induced benign prostatic hyperplasia in rats

**DOI:** 10.3389/fnut.2023.1273299

**Published:** 2023-12-18

**Authors:** Dina Mostafa Mohammed, Tamer M. El-Messery, Denis A. Baranenko, Mahmood A. Hashim, Mohamed Said Boulkrane, Marwa M. El-Said

**Affiliations:** ^1^Nutrition and Food Sciences Department, National Research Centre, Cairo, Egypt; ^2^International Research Centre “Biotechnologies of the Third Millennium”, Faculty of Biotechnologies (BioTech), ITMO University, St. Petersburg, Russia; ^3^Food Technology Research Institute, Agricultural Research Center, Giza, Egypt; ^4^Department of Food and Nutrition, Faculty of Agriculture and Forestry, University of Helsinki, Helsinki, Finland; ^5^Dairy Department, National Research Centre, Cairo, Egypt

**Keywords:** *Phoenix dactylifera L*. seed, dehydrated liposomal, bioaccessibility, soft cheese, benign prostatic hyperplasia, testosterone

## Abstract

**Introduction:**

The consumption of dairy products, including soft cheese, has been associated with numerous health benefits due to their high nutritional value. However, the phenolic compounds bioaccessibility present in soft cheese is limited due to their poor solubility and stability during digestion. So, this study aimed to develop an innovative soft cheese enriched with date seed phenolic compounds (DSP) extracted ultrasonically and incorporated into homogeneous liposomes and study its attenuation effect on testosterone-induced benign prostatic hyperplasia (BPH) in rats.

**Methods:**

Date seed phenolic compounds were extracted using 98 and 50% ethanol along with water as solvents, employing ultrasonication at 10, 20, and 30-min intervals. The primary and secondary DSP-liposomes were prepared and dehydrated. The particle size, zeta potential, encapsulation efficiency, and morphology were measured. Incorporating dehydrated liposomes (1–3% w/w) into soft cheese and their impact on BPH using male Sprague–Dawley rats was assessed. After inducing BPH, rats were fed a cheese diet with dehydrated DSP-liposomes. Over 8 weeks, parameters including nutrition parameters, prostate enlargement analysis, biochemical parameters, hormones level, oxidative stress, and cytokines were analyzed.

**Results and Discussion:**

The results showed that ultrasound-assisted extraction effectively reduced the extraction time and 30 min extraction EtOH 50% was enough to extract high yield of phenolic compounds (558 mg GA/g) and flavonoids (55 mg qu/g) with high antioxidant activity (74%). The biological results indicate that prostate weight and prostate index% were diminished in the treatment groups (1 and 2) compared to the BPH control group. The high antioxidant content present in the DSP-liposomes acted as the catalyst for suppressing the responses of the inflammatory cytokines, inhibiting the anti-inflammatory IL-10 production, and suppressing the elevated levels of lipid peroxidation products compared to the BPH group.

**Conclusion:**

The treatment group (2) supplemented with dehydrated secondary DSP-liposomes exhibited the most significant variance (*p* < 0.05) as opposed to the BPH group. Liposomal encapsulation was proved to be a feasible approach for administering DSP in soft cheese, thereby establishing new functional food category possessing prophylactic properties against the advancement of BPH in rats.

## 1 Introduction

Benign prostatic hyperplasia (BPH) is a non-malignant neoplasm that occurs in males and is associated with aging. The signs and complications of BPH, which include an abnormally enlarged prostate gland, include reduced frequency and flow of urine, difficulty starting the flow, nocturia, and dribbling (1). Prostatic enlargement is often accompanied by distinct changes in tissue histomorphology (2–4). Benign prostatic hyperplasia has been linked to hormone alterations in older men, although the exact cause remains unknown. Dihydrotestosterone (DHT), a biologically active metabolite that synthesizes through the enzymatic conversion of testosterone via steroid 5-reductase, is critical for the androgen stimulation essential to the growth and proliferation of the prostate gland. In the prostate, DHT creation and buildup rise with age, leading to a proliferation of prostate cells and causing hyperplasia (5, 6). Furthermore, benign prostatic hyperplasia results in elevated adrenergic tone in the prostate smooth muscle, which is mediated via 1-adrenoceptors (7).

Biochemical variables contributing to the onset and progression of BPH include an androgen/estrogen ratio that is out of balance and an overabundance of growth hormones (8). The highly lipophilic enzyme 5-reductase, located on intracellular membranes, synthesizes dihydrotestosterone (DHT), a testosterone metabolite, and a key modulator of prostate development, in the prostate from circulating testosterone (9). The destruction of essential tissue components, including DNA, messenger RNA (mRNA), and proteins, may be attributed to oxidative stress, which performs a pivotal function in prostatic hyperplasia by generating a contradiction between free radical production and elimination (10). Growth factors, cytokines, and steroid hormones, which promote cell proliferation and reduce cell death, affect the contradiction between prostate cell development and apoptosis (11).

Date seed phenolic compounds offer potential antioxidant, anti-inflammatory, antimicrobial, and neuroprotective benefits. They may support cardiovascular health, aid in diabetes management, promote liver health, and could have anti-cancer properties (12, 13). These properties may help to attenuate the development of BPH by reducing inflammation and oxidative stress in the prostate gland (14). The phenolic compounds that may be beneficial for BPH include phenolics, tannins, and flavonoids, which found in many fruits, vegetables, and seed, and has been shown to have anti-inflammatory properties. Gallic acid, protocatechuic acid, p-hydroxybenzoic acid, vanillic acid, caffeic acid, p-coumaric acid, ferulic acid, m-coumaric acid, and o-coumaric acid have been identified in Mabseeli date seed (15), and has been shown to have anti-inflammatory, anti-cancer properties, and BPH (16, 17). Ultrasound-assisted extraction (UAE) is considered an environmentally friendly extraction process that can yield higher amounts of phenolic compounds compared to conventional and alternative non-conventional extraction techniques (18, 19). The aforementioned technique is characterized by its simplicity, affordability, expeditiousness, and efficacy in the isolation of bioactive constituents from botanical specimens (20). Additionally, UAE has been utilized as a means to extract phenolic compounds from the seed of dates (*Phoenix dactylifera* L.) (21, 22).

The primary drawbacks of palm seed polyphenol extract powder include its low-solubility aqueous phase, unpleasant taste, susceptibility to high temperature, destruction during food manufacturing, alkaline conditions, and storage. So, the bioaccessibility of these compounds is limited.

Liposomes have been identified as a viable encapsulation technique for effectively delivering phenolics. Additionally, liposomes possess the desirable characteristics of being biodegradable, biocompatible, and non-immunogenic, thereby exhibiting significant potential for utilization in the pharmaceutical, food, and agricultural sectors as well as to overcome the limitation of these bioactive compounds bioaccessibility (23–25). Applying a polymer material to the surface of liposomes is a viable approach for ensuring the preservation of their stability. Protective coatings such as chitosan, pectin, or a combination of both can be utilized in the acidic environment of the stomach and as triggered release systems in the colon (26). However, utilizing such fluid systems in food formulations poses a considerable challenge. The study conducted by El-Messery et al. (27), El-Said et al. (28), and Moraes et al. (29) demonstrated that the feasibility of utilizing liposome-encapsulated phenolics in food formulation was enhanced upon drying, which in turn worked to improve their physicochemical stability and storage properties and enhanced their *in vitro* bioaccessibility.

The consumption of dairy products, including soft cheese, has been associated with numerous health benefits due to their high nutritional value (30). However, the bioaccessibility of certain bioactive compounds present in soft cheese, such as phenolic compounds, is limited due to their poor solubility and stability during digestion (31, 32). This limits their potential health benefits and may contribute to the development of chronic diseases (33). So, this research aims to investigate the use of date seed phenolic-loaded dehydrated liposomes to enhance the bioaccessibility of phenolic compounds in soft cheese is a novel approach that has the potential to improve the nutritional value and health benefits of dairy products. Additionally, the investigation of the potential attenuation effect of this delivery system on testosterone-induced BPH in rats provides valuable insights into the therapeutic potential of phenolic compounds in the prevention and treatment of this condition.

## 2 Materials and methods

### 2.1 Materials

The investigation employed three cultivars of date seed, namely “Medjool, Amri, and Siwi,” procured from the Central Laboratory for Palm Research and Development in Giza. Fresh ultrafiltration buffalo cream retentate was provided by the Animal Production Research Institute (Agriculture Research Center, Dokki, Egypt). The retentate obtained had the following composition: 21.9% total solids, 15.4% protein, 1.5% fat, and 3.7% lactose. The commercial rennet powder used was Valiren 150, a microbial cheese rennet obtained from Valley Research, Inc. (IN, United States). The rennet powder had a concentration of 1:150,000 Mcu/g. The soy lecithin granules utilized as a nutritional supplement were obtained from Solgar Company (Leonia, NJ, United States). The source of the soybean used to derive the product was confirmed to be a non-genetically modified organism (GMO). The enzymes, analytical and lab-grade chemicals, and solvents, as well as the testosterone used in the study were procured by Sigma Chemical Company (Sigma-Aldrich Co., St. Louis, United States). The kits for quantitative analysis were provided by Fine Test (Wuhan Fine Biotech Co., Ltd.; Wuhan, China), Sunlong Biotech Co. (Yuhang, China), and Biodiagnostic Company (Egypt).

### 2.2 Preparation of date seed powder

The preparation of date seed powder involved 10 kg of fully ripe date fruits. The seed were rinsed, oven-dried, and ground using a cutting mill—Retsch SM 100 with final fineness due to bottom sieve with aperture size 1 mm. This powder was then used for extraction and subsequent analysis.

### 2.3 Ultrasound-assisted extraction of date seed powder

To extract bioactive compounds from date seed powder it was mixed at a ratio (1:10) with ethanol (EtOH) 98%, EtOH 50%, and water. The mixture was then sonicated (Sonics Vibra Cell sonicator at 160 W powers, 20 kHz frequency, and 50% pulse) for 10, 20, and 30 min with controlling the temperature by surrounding the beaker with ice. A control sample extracted by agitation for 2 h using EtOH 50% as a solvent was used. Eliminating ethanol involved using a rotary evaporator (BÜCHI Labortechnik AG, Flawil, Switzerland) at 40°C under reduced pressure until its complete removal. The residual aqueous extract was treated in a freeze drier (Labconco Co., Kansas City, United States) at a temperature of −52°C for 48 h under a vacuum pressure of 0.1 mPa. The powder obtained was stored at a temperature of −18°C until analysis.

### 2.4 Determination of total phenolic content, total flavonoid content, and total antioxidant activity

The Folin–Ciocalteu reagent was used to measure the samples’ total phenolic content (TPC), according to the methods outlined by Zaky et al. (34). Based on the calibration curve, the total phenolic content was quantified, and the results were expressed in mg of gallic acid (GA) equivalent per gram of the sample.

The total flavonoid content (TFC) was assessed using the aluminum chloride colorimetric method, as described by Nurcholis et al. (35). The flavonoid concentration per gram of sample was quantified by employing the quercetin (qu) solution calibration standard. The resulting values were expressed in mg of quercetin equivalent (qu) per gram of the sample.

The method described to evaluate the proportion (%) of DPPH free radical scavenging activity by Zaky et al. (36) was used.

### 2.5 Preparation and characterization of dehydrated primary and secondary DSP-liposomes

A dispersion of lecithin (2%, w/w) was formulated in an acetate buffer with a pH of 3.5 ± 0.1 and a concentration of 0.1 M. A solution containing DSP with a concentration of 0.6% (w/v) was then dissolved in a lecithin solution. The primary DSP-liposome was obtained through probe sonication, using an instrument from Sonics and Materials, Inc. Vibra-Cell with a power of 160 W, a frequency of 20 kHz, and a 50% pulse. Secondary liposomes were prepared using the layer-by-layer deposition technique by stirring the primary DSP-liposomes in chitosan (0.4%, w/v) dissolved in acetate buffer solution (pH = 3.5 ± 0.1; 0.1 M) at room temperature for 12 h. The multilayered liposomes (primary and secondary DSP-liposomes) were combined with maltodextrin (20%) in a 1:1 (v/v) ratio and agitated for an extended peri (2 h) at room temperature. The multilayered liposomes (primary and secondary DSP-liposomes) were dried by freeze-drying using a laboratory freeze dryer at a temperature of −52°C for 48 h under a vacuum pressure of 0.1 mPa and were then stored at a temperature of −18°C until used.

The dehydrated primary and secondary DSP-liposomes were characterized by assessing their ζ-potential and particle size distribution. The ζ-potential was measured with a particle charge titration analyzer (Stabino®, Microtrac Europe, and Montgomeryville, PA, United States). The particle size distribution was analyzed with the Mastersizer MS2000 (Malvern Instruments, Worcestershire, United Kingdom).

The encapsulation efficiency (EE) of dehydrated primary and secondary DSP-liposomes was determined with the method outlined by González-Ortega et al. (37). The pellet containing liposomes were centrifuged at a force 12,000 *g* for 180 min at a temperature of 20°C. The presence of unencapsulated phenolic compounds was checked in the resultant supernatant. To quantify the encapsulated phenolic compounds, a mixture of 1 mL methanol and 1 mL chloroform (1:1, v/v) was used to disrupt the liposome pellets that have been resuspended. Prior to phase separation, the mixture underwent vigorous vortexing. Phenolic concentrations were quantified in the aqueous-methanolic top layer, and the encapsulated and non-encapsulated phenolics were determined. EE was determined using Eq. (1):


(1) $$EE\;\left(\%\right)=\frac{TPC-FPC}{TPC}X10$$

TPC: Total phenolic compounds (encapsulated and non-encapsulated).

FPC: Free phenolic compounds (non-encapsulated).

The scanning electron microscope in the study is the FEG 250 (FEI, United States). Before the analysis, the samples were coated with gold sputter using a Leica vacuum coating unit to mitigate the samples charging. The SEM images display the information regarding the operating conditions, including the accelerating voltage, magnification, and working distance.

### 2.6 Soft cheese manufacturing

Cheese was manufactured using ultrafiltration retentate, according to Soliman et al. (38) and Hala et al. (39) methods. The retentate was then pasteurized into a glass jar that had been maintained at 72°C for 15 s, and cooled to 4°C, then regulated to a temperature of 42°C. It was further divided into four portions. The dehydrated secondary DSP-liposome with the highest EE was added to the retentate samples at the ratios of 1, 2, and 3% (w/w) for T1, T2, T3, and a control cheese sample without the addition of liposome, respectively. Then the rennet was added to the glass jars in the ratio 3 g/100 L retentate at 37°C with stirring for 1 min, followed by incubation at a temperature of 42°C until complete coagulation, which took approximately 40 min. The cheese samples were refrigerated at a temperature of 5 ± 2°C. Three replicates originating from each portion were prepared and analyzed.

### 2.7 Cheese analysis

The chemical composition of cheese samples was assessed by the guidelines from AOAC (40). The pH levels of cheese samples were assessed with a Jenway 3510 pH meter. The texture measurement parameters of cheese samples, including cohesion, hardness, springiness, gumminess, and chewiness were assessed using the dual stress test (TMS-Pro Texture Analyzer, United States) of Zheng et al. (41).

### 2.8 *In vitro* digestion

To evaluate the bioaccessibility (%) of encapsulated DSP loaded liposomes in soft cheese, Tan et al. (42) subjected the samples to simulated conditions of gastrointestinal tract during digestion. First, a basal saline solution with a volume of 13.5 mL, containing 140 mM NaCl and 5 mM KCl, was mixed with 1.5 mL of the sample. After that, the mixture was shaken for 10 min at 400 rpm in a water bath at 37°C (New Brunswick Scientific Co., Inc., New Jersey, United States). The mixture was further exposed to 4.5 mL of simulated gastric fluids that contained 3.2 g/L pepsin in 1 M HCl. Gastric digestion was initiated by adjusting the pH to 2.0 with 1.0 M NaOH. After an incubation period of 1 h at a temperature of 37°C, the intestinal digestion was initiated by adjusting the pH of the samples to 7.5 by adding 1.0 M NaOH. Then, a volume of 4.5 mL of simulated intestinal fluid consisting of a phosphate buffer solution containing pancreatin at a concentration of 4.76 mg/mL and porcine bile extract at a concentration of 5.16 mg/mL was added. After 2 h of the intestinal digestion, the collected samples were centrifuged at a rate of 6,000 rpm for 15 min. The uppermost phases were gathered and preserved at −20°C for subsequent assessments. The total phenolic and flavonoid content, as well as antioxidant activity were evaluated according to Section 2.3., the bioaccessibility (%) calculation was performed by Equation (2):


(2) $$\mathrm{Bioaccessibility}\left(\%\right)=\frac{\mathrm{AmountofDSPdetectedafterdigestion}}{\mathrm{AmountofDSPdetectedbeforedigestion}}\times 100$$

### 2.9 Animal study

Male Sprague–Dawley rats acquired from the National Care Unit, NRC, Cairo, Egypt, were 12 weeks old. Moreover, it weighed between 240 and 260 g. In Egypt’s National Research Centre, all rats received care under the Animal Experimental Guidelines, which the Medical Research Ethical Committee approved (Approval No. 7411062021) under the United Kingdom’s Animals (Scientific Procedures) Act, 1986, and associated guidelines, EU Directive 2010/63/EU for animal experiments (Publication No. 85–23, revised 1985). Prior to the commencement of the study, the animals were provided with a standard laboratory diet along with free access to water and housed in plastic cages for approximately 7 days. This enabled the animals to acclimate and ensure they would develop and behave normally. The housing room was operated at 24°C with a relative humidity of 40–60%, lighting (12 h of darkness followed by 12 h of light), and was pollutant-free.

### 2.10 Diet formula

A basic balanced diet is containing various essential components as casein (150 g/kg), unsaturated fat (100 g/kg), sucrose (220 g/kg), maize starch (440 g/kg), cellulose (40 g/kg), salt mixture (40 g/kg), and vitamin mixture (10 g/kg) (43, 44). The salt and vitamin mixtures were formulated in line with the AIN-93 M diet (45).

### 2.11 Model of benign prostatic hyperplasia

The prostate was over-stimulated in a BPH rat model via subcutaneous injection of testosterone propionate in corn oil (10 mg/kg/day) for 8 weeks after castration via aseptic excision of both the testes (46). For 7 days after castration, the rats were kept in a controlled environment to ensure complete involution of the prostatic gland.

### 2.12 Experimental design

The present research involved 24 rats/four groups, each with six rats as follows:

- Normal control: rats were daily fed the essential balanced diet and were orally administered with phosphate-buffered saline (PBS) in corn oil.
- BPH group: rats were administered with testosterone propionate subcutaneously (10 mg/kg/day) and were daily fed the essential balanced diet for 8 weeks.
- Treatment group (1): rats were administered with testosterone propionate subcutaneously (10 mg/kg/day) and were fed a daily essential balanced diet supplemented with soft cheese containing dehydrated primary DSP-liposomes (600 mg/kg BW/day) (47).
- Treatment group (2): rats were administered with testosterone propionate subcutaneously (10 mg/kg/day) and were fed a daily essential balanced diet supplemented with soft cheese containing dehydrated secondary DSP-liposomes (600 mg/kg BW/day) (47).

Seven days following castration, all treatments were administered in conjunction with testosterone. After the 8-week experimental period euthanasia was applied to rats using an intramuscular ketamine hydrochloride injection (35 mg/kg). Twelve hours of fasting preceded the cervical dislocation and euthanasia of all the animals. All nutritional parameters were tracked throughout the research period.

### 2.13 Preparation of blood samples

Tail vein blood was obtained from each animal and then centrifuged for approximately 15 min at 4,000 rpm to separate the serum and plasma using the centrifuge from Sigma Labor Centrifuge GMBH, West Germany (model 2-153360 Osterode/Hertz). For the analysis of sex hormones, fresh serum was used immediately after collection, but it was otherwise maintained at −80°C until required for biochemical assays.

### 2.14 Evaluation of prostate enlargement

#### 2.14.1 Prostate weight (g)

The prostate was gently removed, collected, and promptly weighed.

#### 2.14.2 Prostate index %

The prostate index (%) = the weight of prostate (g)/the weight of body (g) × 100.

### 2.15 Preparation of prostate homogenates for tissue analysis

The prostate tissue was homogenized into a 10% homogenate in an ice-cold 50 mM potassium phosphate buffer with a pH of 7.4. The homogenate was split into supernatant and preserved at −80°C until used in all subsequent assays following centrifugation at 15,000 *g* for duration of 30 min at a temperature of 4°C (38).

### 2.16 Estimation of testosterone, dihydrotestosterone, and caspase-3 levels

The testosterone and DHT hormones, as well as caspase-3 were evaluated in prostate tissues using ELISA kits (MyBioSource, CA, United States) following the manufacturer’s instructions.

### 2.17 Cytokines quantitation

The cytokines including interleukins (IL-6, IL-10, and IL-1β) and the tumor necrosis factor-α (TNF-α) were evaluated by ELISA kits (Sunlong Biotech, China) following the manufacturer’s instructions.

### 2.18 Determination of oxidative stress markers

Malondialdehyde (MDA), reduced glutathione (GSH), superoxide dismutase (SOD), catalase (CAT), glutathione peroxidase (GPx), and glutathione reductase (GR) were evaluated spectrophotometrically using the methods of Satoh (48); Ellman (49); Kakkar et al. (50); Sinha et al. (51); Rotruck et al. (52); and Goldberg (53), respectively.

### 2.19 Determination of lipid profiles, hepatic function biomarkers, and kidney function biomarkers

Total cholesterol, HDL, LDL, and triglycerides were evaluated spectrophotometrically using the enzymatic colorimetric techniques (54–57). Plasma alanine aminotransferase (ALT), aspartate aminotransferase (AST), and alkaline phosphatase (ALP) were assessed spectrophotometrically using colorimetric assays (58, 59). Creatinine and urea were analyzed spectrophotometrically using colorimetric techniques (60, 61).

### 2.20 Statistical analyses

Statistical data analysis was conducted using SPSS (IBM® SPSS®, 2017). The mean values and the standard deviation were computed. ANOVA and Duncan’s multiple range tests were applied to examine differences at a 5% significance level (*p* < 0.05).

## 3 Results and discussion

### 3.1 Total phenolic content, total flavonoid content, and total antioxidant activity of date seed extracts

**Figure 1:**
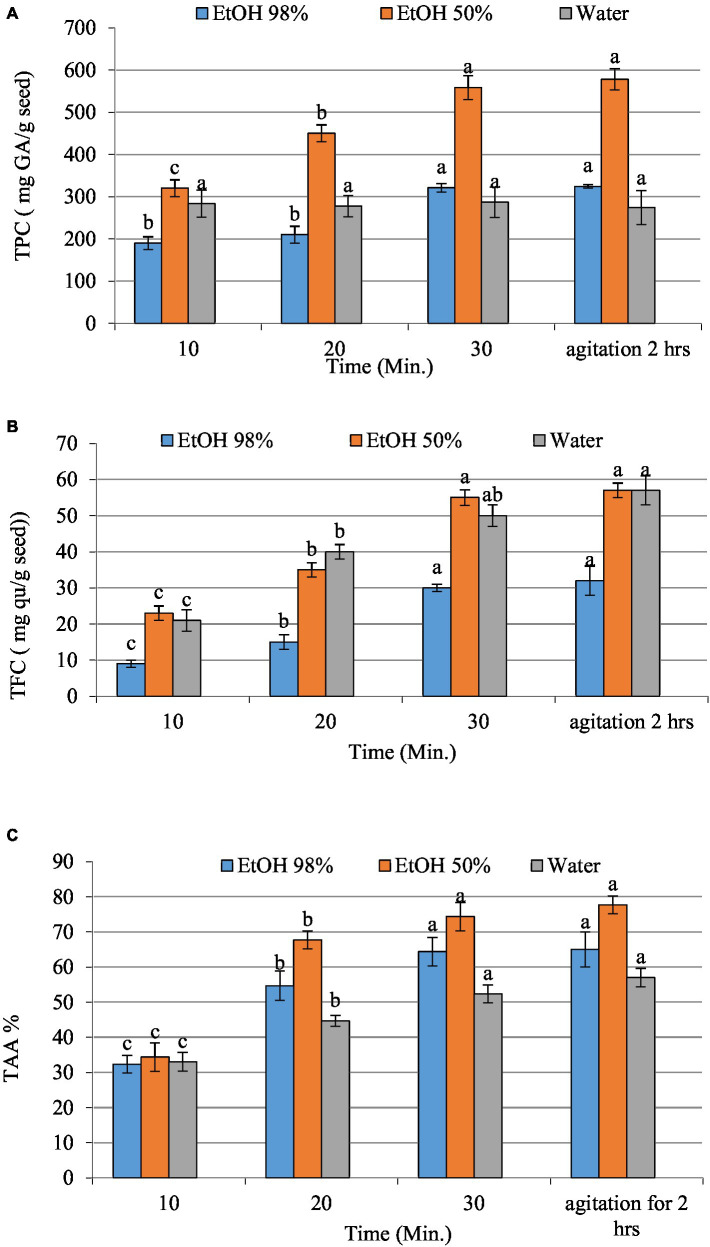
The total phenolic **(A)**, total flavonoids content **(B)**, and total antioxidant activity **(C)** of date seed extracts (EtOH 98 and 50% and water). Values with different letters on the same samples represent statistical differences according to a one-way ANOVA (*p* < 0.05).

Figure 1 illustrates TPC, TFC, and TAA of date seed extracts obtained using various solvents, including ethanolic solvents with 98 and 50% concentrations, and water, at different ultrasonication times (10, 20, and 30 min) and agitation for 2 h using EtOH 50% as a control. The findings suggest that using 50% EtOH resulted in the highest phenolic and flavonoid content with the highest antioxidant activity compared to 98% ethanol and water used as solvents. Besides, a higher duration of ultrasonication affected the extraction of phenolic compounds and antioxidant activity. In particular, the results revealed that the total phenolic content (TPC), total flavonoid content (TFC), and total antioxidant activity significantly increased after 30 min of ultrasound-assisted extraction. On the other hand, there was no significant difference observed when compared to the control group subjected to 2 h of agitation with 50% EtOH. These results indicate that ultrasound-assisted extraction effectively reduced the extraction time while maintaining or even enhancing the extraction efficiency of phenolic compounds. This may be attributed to the high concentration of polyphenols being released. These findings are consistent with the research conducted by Salomón-Torres et al. (62) who reported that the seed of “Medjool” dates has polyphenolic content 10 times greater than that found in the pulp. The study by Saleh et al. (63) revealed a similar trend; they claimed that the date palm may contain water-soluble phenolic compounds.

Numerous research studies have investigated the biological properties of phenolic compounds, which exhibit robust antioxidant and free radical scavenging capabilities; it indicates that the combination of ethanol and water (50:50) exhibits a notable capacity for antioxidant activity and scavenging of free radicals (1.26 ± 0.02 μmol Trolox/g) (64, 65). The results showed that the extract derived from date seed possesses strong antioxidant properties in ethanol/water extract. Present study findings seem to concur with the study by Saleh et al. (63) who argued that date fruits are abundant in hydrophilic antioxidants, and their reducing potential is mainly attributed to polyphenols. Halabi et al. (66) and El Sohaimy et al. (67) have demonstrated a direct correlation between antioxidant activity and the total phenolic content in dates. The findings of the current investigation indicate that Egyptian date seed possess a substantial amount of phenolic compounds and exhibit a high antioxidant potential. This suggests they may be a viable option for functional food applications. The most effective treatment, as shown by our research, involves applying ultrasound for 30 min and 50% ethanolic extraction solution. Consequently, it is these parameters that were employed in the formulation of liposomes.

### 3.2 Characterization of dehydrated primary and secondary DSP-liposome

Table 1 shows the mean particle size of the dehydrated primary and secondary DSP-liposome. This analysis indicates that the incorporation of DSP into liposomes resulted in a significant increase in the mean particle size of the powdered primary liposome (355.25 ± 3.61 nm) and (145.18 ± 2.26 nm). This aligns with the findings outlined by Alshafei et al. (68) who employed a comparable methodology to encapsulate red beet peel extract in liposomes. El-Messery et al. (20) conducted a study on integrating encapsulated mandarin peel extract in liposome system and incorporating it in processed cheese, while El-Said et al. (21) encapsulated doum extract in soy phospholipids liposomes. Both studies revealed that soy phospholipids at a concentration of 2% resulted in an augmentation of the liposome particle size. The higher dimensions of liposomal particles are a common occurrence that results from their susceptibility to modification caused by the aggregation of labile liposomes during both the processing and preservation stages (69). The ratio of bioactive substances to phospholipids is a crucial determinant that can impact the dimensions of a colloidal entity, such as liposomes. The size of liposomes can be increased by increasing the concentration of active substances. As a result, the ratio of the core to the membrane is decreased. This phenomenon can be attributed to the active materials occupying a significant portion of the liposomes, as reported by Sebaaly et al. (70, 71). Introducing a cationic polymer, namely chitosan, into the compositions resulted in a minimum four-fold increase in the dimensions of the particles, as indicated in Table 2.

**Table 1:** Mean particle size, ζ-potential, and EE of dehydrated primary and secondary DSP-liposomes.

Type of liposome	Particle size (nm)	ζ-potential (mV)	Encapsulation efficiency (%)
Empty liposome	145.18 ± 2.26^c^	−25.20 ± 1.34^b^	-
Primary DSP-liposome	355.25 ± 3.61^b^	−28.33 ± 1.66^a^	82.43 ± 1.79^b^
Secondary DSP-liposome	1756.85 ± 1.63^a^	28.54 ± 1.32	90.36 ± 1.26^a^

Values are the means of three replicates ± SD. Various superscript letters are used to indicate significant differences among samples for each parameter within the same row (*p* < 0.05).

**Table 2:** The physicochemical properties and texture profile of soft cheese enriched with different concentrations of dehydrated secondary DSP-liposomes.

Parameters	Control (without liposome)	T1	T2	T3
Moisture (%)	69.66 ± 0.11^a^	68.72 ± 0.35^b^	66.33 ± 0.30^c^	65.35 ± 0.29^d^
Dry matter (%)	30.35 ± 0.11^d^	31.29 ± 0.35^c^	33.68 ± 0.30^b^	34.66 ± 0.29^a^
Protein (%)	11.21 ± 0.06^a^	11.22 ± 0.13^a^	11.17 ± 0.04^a^	11.18 ± 0.13^a^
Fat (%)	10.18 ± 0.08^a^	10.20 ± 0.07^a^	10.13 ± 0.06^a^	10.22 ± 0.08^a^
Ash (%)	2.79 ± 0.03^a^	2.81 ± 0.01^a^	2.79 ± 0.01^a^	2.80 ± 0.04^a^
pH	5.64 ± 0.01^a^	5.61 ± 0.01^a^	5.57 ± 0.01^b^	5.54 ± 0.01^b^
Hardness (*N*)	12.62 ± 1.32^c^	14.21 ± 2.02^bc^	17.20 ± 0.65^ab^	18.58 ± 1.26^a^
Springiness (mm)	0.72 ± 0.02^a^	0.71 ± 0.01^a^	0.75 ± 0.01^a^	0.71 ± 0.01^a^
Cohesiveness	0.64 ± 0.02^a^	0.64 ± 0.01^a^	0.66 ± 0.01^a^	0.65 ± 0.04^a^
Gumminess (*N*)	7.50 ± 0.07^a^	7.44 ± 0.18^a^	7.40 ± 0.06^a^	7.49 ± 0.09^a^
Chewiness (N*mm)	5.15 ± 0.04^c^	5.23 ± 0.04^bc^	5.32 ± 0.02^a^	5.27 ± 0.02^ab^

Values are the means of three replicates ± SD. Various superscript letters are used to indicate significant differences among samples for each parameter within the same row (*p* < 0.05). Control sample: without liposomes; T1: soft cheese enriched with 1% (w/w) of dehydrated secondary DSP-liposomes; T2: soft cheese enriched with 2% (w/w) of dehydrated secondary DSP-liposomes; and T3: soft cheese enriched with 3% (w/w) of dehydrated secondary DSP-liposomes.

As shown in Table 1, the ζ-potential analysis, an observable transition in the ζ-potential of the structures is evident, manifesting a shift from negative to positive values. This transition is particularly pronounced when examining the dehydrated primary DSP liposomes, which reveal a charge of −28.7 mV, in contrast to the dehydrated secondary DSP-liposomes that exhibit a charge of 26.5 mV. These findings underscore the introduction of a positively charged chitosan layer onto the surface of liposomes in both instances. Two crucial parameters for achieving successful surface coating of liposomes are the particle size increase and surface charge modification. The augmentation of zeta potential results in a concomitant increase in the repulsive force among the particles, thereby impeding their collision and subsequent accumulation. Conversely, increasing the charge of particles enhances their interaction with the recipient cells, thereby elevating the transportation of the bioactive agent(s). Samples exhibiting low ζ-potential possess a marginal repulsive force, leading to particle binding and consequent physical instability, as Heurtault et al. (72) reported. For colloidal particles to exhibit resistance to electrostatic repulsion, it is typically necessary for the ζ-potential of the entire structure to exceed a value of ±30 mV. In prior research, it has been documented that the incorporation of a phenolic extract with a negative charge into an anionic liposomal dispersion results in the elevation of the overall negative zeta potential of the liposomal dispersions (20, 21, 73).

Encapsulation efficiency is of particular importance for liposome formation and is affected by several variables, including the coating material used (lipophilic or hydrophilic materials), the concentration and the composition of phospholipids, the process of preparation, and environmental factors such as pH and temperature. Table 1 gives EE values for dehydrated primary and secondary DSP-liposomes. The findings of the current investigation demonstrated a noteworthy enhancement in EE (90.36 ± 1.26%) of the powdered secondary liposomes compared to the powdered primary liposomes (82.43 ± 1.79%). Several studies have suggested that an elevation in phenolics concentration could potentially aid in the improvement of liposome EE. Thus, a dehydrated secondary DSP-liposome was incorporated in the cheese (74–77).

**Figure 2:**
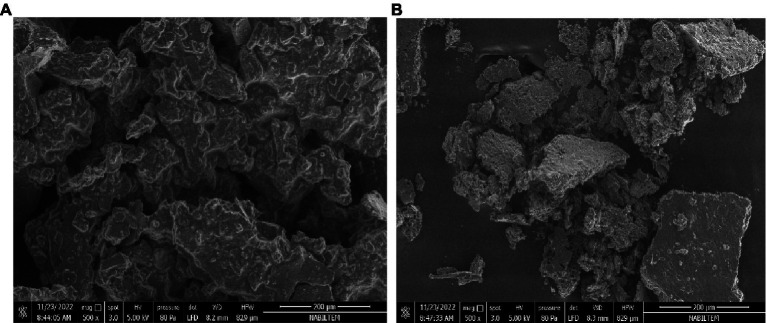
Morphology of dehydrated primary **(A)** and secondary **(B)** DSP-liposomes.

Figure 2 shows SEM images of discrete dehydrated primary (A) and secondary (B) DSP-liposomes in powdered form. The surfaces exhibit wrinkles depicted in Figure 2, which can be attributed to the rapid shrinkage of the sample during the cooling phase of the freeze-drying process. Moreover, the topography of the liposome loaded with DSP that underwent freeze-drying exhibited irregularities and lacked homogeneity. This phenomenon could be attributed to lyophilization. The dehydrated primary DSP-liposome had a smoother surface morphology compared to the dehydrated secondary DSP-liposomes, which suggests that chitosan may have played a role in drying it (Figures 2A,B). Furthermore, chitosan may have contributed to the formation of wrinkled surfaces, as suggested by Guldiken et al. (78), as it is a potential wall material. The incorporation of chitosan has been observed to impact the product’s morphology, resulting in the formation of a cross-link with lecithin and the development of a dense and irregular structure. This suggests a negligible probability of core material escaping to the environment and proves that the liposomes were properly prepared.

### 3.3 Physicochemical properties of soft cheese enriched with dehydrated secondary DSP-liposomes

Table 2 displays the chemical composition and the pH of cheese samples enriched with varying concentrations (0, 1, 2, and 3% w/v) of dehydrated secondary DSP-liposomes, as measured on the day following their production. In terms of composition, Table 2 indicates that no statistically significant disparities (*p* > 0.05) were observed between the control enriched cheese samples with DSP-liposomes, except for the moisture and dry matter, the former decreasing and the latter decreasing. The results obtained from the present investigation indicate that the incorporation of dehydrated secondary DSP-liposomes into cheese had almost no effect cheese sample chemical composition. The observed decrease in moisture ratio of cheese samples after incorporating 1% secondary DSP-liposome is in accordance with Al-Moghazyet al (79). results who reported a similar effect in Karish cheese upon liposome addition. The researchers claimed that this phenomenon may be attributed to the liposome membranes’ ability to adhere to water at their outer layer, which can be preserved in the cheese composition. The study conducted by Siyar et al. (80) revealed that incorporating encapsulated saffron extract (SE) in liposome to fortify ricotta cheese had no adverse effect on the product chemical composition. Moreover, a marginal rise in the dry matter proportion was observed. Lecithin, which exhibits hydrophilic and hydrophobic characteristics, can interact with fat and moisture (81). Hence, incorporating lecithin as a fundamental constituent in the liposomal formulation could contribute to the elevation of moisture content in the specimens enriched with greater concentrations of liposomes, as observed in this investigation. On the other hand, as mentioned above, the technique did not produce any significant alterations in the soft cheese composition.

Cheese sample pH levels were assessed, and the results are presented in Table 2. The findings suggest that there is a statistically negligible difference (*p* > 0.05) between the control sample and the cheese samples that contain secondary DSP-liposome powder up in a concentration of 1% and lower. The control sample exhibited the highest pH value among the fortified cheese samples, whereas the cheese enriched with 3% dehydrated secondary DSP-liposome exhibited the lowest pH. This finding does not align with the anticipated effect of increased moisture content in the liposome-containing samples. The observed phenomenon can be attributed to the positive correlation between elevated moisture levels and the generation of fatty acids, as reported by Aminifar et al. (82). The marginal decline in pH after introducing liposomes may be attributed to acidic constituents (acetate buffer with a pH of 3.8) within the dehydrated secondary DSP-liposome formulation. In Jeong et al. (83), the physicochemical properties of Queso Blanco cheese were investigated by supplementing powdered microcapsules of tomato extract. Their findings indicated a decrease in cheese pH, consistent with their previous research (83). The authors suggest that the acidic pH of bioactive compounds may contribute to reducing pH in the medium to which they are added.

Texture Profile Analysis (TPA) is a valuable indicator for assessing the texture attributes of cheese (84). The present investigation TPA of control cheese samples and those fortified with varying concentrations of dehydrated secondary DSPE-liposome powder. The TPA was performed to evaluate the samples’ hardness, adhesive properties, cohesiveness, gumminess, and chewiness. The results of the TPA are presented in Table 2. Overall, the texture of the cheese is contingent upon its microstructure and chemical composition, with particular emphasis on factors such as fat, salt, and overall solids concentration (85). Table 2 illustrates that only statistically negligible variations (*p* > 0.05) were observed in the adhesiveness, cohesiveness, or gumminess for all cheeses. However, the soft cheese samples that contained dehydrated secondary DSP-liposome exhibited a significant increase in hardness and chewiness. Jeong et al. (83) reported that the texture of Queso Blanco cheese was influenced by its pH. Their study found that a decrease in pH after adding microcapsules containing lycopene extract in high concentrations increased the cheese hardness, gumminess, and chewiness. During curd formation, the protonation of ionic types of molecules that form covalent bonds with casein occurs as the pH decreases. The increased hydrophobic interactions among protein molecules result in an increase in the hardness of the curd (86). The hardness of cheese derived from milk coagulated at pH 6.5 was more pronounced than of that produced at pH 6.1 (84). The present investigation suggests a similar phenomenon may occur when fortifying cheese with encapsulated secondary DSP-liposome compared to the control sample, as evidenced by the lower pH levels observed in the enriched cheese samples. Cheese texture depended on its fat content. Additionally, using milk fat in the production of ricotta cheese was observed to enhance its adhesiveness (87). However, the results differed from the findings of Rubel et al. (88). They reported an inverse relationship between the fat content of cheese and its adhesiveness. The texture of reduced-fat ricotta cheese was firmer compared to that ofricotta cheese with a high fat content (89). Nonetheless, the outcomes observed in the earlier investigations are not generalizable to the findings of our current study due to the absence of a noteworthy dissimilarity in the lipid composition between the liposomal-enriched cheese samples and the control one, as indicated in Table 2. Lecithin is a fundamental and essential component within the structural framework of liposomes. Adding lecithin at 0.5% to reduced-fat cheese resulted in hard texture. This was attributed to the interactions between lecithin and casein. Upon analyzing the microstructure, the investigators noted a significant disruption in the casein. The moisture in the cheese produced textural defects (81). However, at reduced concentrations, moisture ratio in cheese did not seem to impact its protein, thereby preserving the customary texture of the cheese. The present study showed that the addition of lecithin did not result in any significant changes in the fat content of the cheese samples. However, it is plausible that the inclusion of lecithin may have played a role in the increased gumminess and chewiness of the cheese samples that were enriched with dehydrated secondary DSP-liposomes. The liposomes improved the flavor and mouthfeel of the cheese matrices. The mechanism of interaction was attributed to the formation of a network of liposomes and proteins, which acted as a carrier for flavor compounds and improved the texture of the cheese matrix (90).

### 3.4 *In vitro* digestion

**Figure 3:**
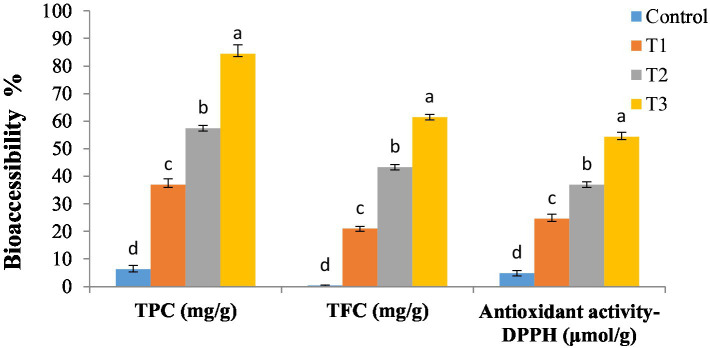
*In vitro* bioaccessibility of secondary DSP-liposomes in soft cheese. Control sample: without liposomes; T1: soft cheese enriched with 1% (w/w) of dehydrated secondary DSP-liposomes; T2: soft cheese enriched with 2% (w/w) of dehydrated secondary DSP-liposomes; and T3: soft cheese enriched with 3% (w/w) of dehydrated secondary DSP-liposomes. Values with different letters on the same property represent statistical differences according to a one-way ANOVA (*p* < 0.05).

The present study employed an *in vitro* gastrointestinal digestion model to assess the TPC, TFC, TAA, and bioaccessibility (%) of DSP in secondary liposomes integrated in soft cheese (T1, T2, and T3; as shown in Figure 3). The results indicated that the bioaccessibility of the control sample was low compared to that of the enriched cheese samples containing DSP-liposome. Furthermore, a significant enhancement in the bioaccessibility of DSP was observed with an increase in the proportion of DSP-liposomes incorporated into cheese, with T3 exhibiting the highest bioaccessibility followed by T2 and T1. The bioaccessibility of TPC and TFC was observed to increase by a minimum of six times and eight times, respectively, upon encapsulation in secondary liposomes. The results of the DPPH assay indicate a 4-fold increase in antioxidant activity. The inclusion of MD in liposomes, in addition to chitosan, may improve the stability of phenolic compounds in the gastrointestinal environment. The observed phenomenon can be attributed to the surface modification of liposomes with cationic chitosan, which increased their *in vitro* bioaccessibility. Incorporating a polymer layer onto the liposome surface facilitated the elimination of both enzymatic and acidic degradation of DSP located on the liposome surface during the gastric phase (75).

The interaction between phospholipids and maltodextrin has been studied in various contexts, including liposome formation and stabilization. The maltodextrin increased the stability of liposomes and prevented their aggregation and fusion. The mechanism was attributed to electrostatic and hydrophobic interactions between the hydrophilic head groups of the phospholipids and hydroxyl groups of maltodextrin (91). The maltodextrin improved the stability of liposomes and increased the bioaccessibility of drugs (a bioactive compound with poor water solubility) (92). On the other hand, the mechanism of interaction was attributed to the formation of hydrogen bonds between the hydroxyl groups of the maltodextrin and the phosphate groups of the phospholipids (93). Liposomes improved the texture and reduced the syneresis of cheese matrices. The mechanism of interaction was explained by the formation of a network of liposomes and proteins, which stabilized the cheese matrix and prevented the release of moisture and volatile compounds (94). Thus, the interaction between phospholipids and maltodextrin, and between liposomes and cheese matrices, can involve multiple mechanisms including electrostatic and hydrophobic interactions, as well as formation of networks between liposomes and proteins. These mechanisms can improve stability, bioaccessibility, texture, and flavor of the final product.

### 3.5 Animal study results

#### 3.5.1 Growth-related parameters

The nutritional results of an 8-week study were illustrated in Table 3, which compares the normal control, BPH, and treatment groups (1, 2) in terms of initial and final body weights, body weight gain, total food intake, and feed efficiency. The results showed no significant initial body weights across the experimental groups (47, 95, 96). When comparing the BPH group to the normal control, a significant decline in final body weights, weight gain, total food intake, and feed efficiency was observed (97). Comparing the treatment groups (1 and 2) to the BPH control significantly increased final body weights, weight gain, and total food intake.

**Table 3:** Effects of soft cheese containing dehydrated primary and secondary DSP-liposomes on nutritional parameters and prostate profile.

Groups	Normal control	BPH control	Treatment group (1)	Treatment group (2)
Parameters				
Initial body weight (g)	245 ± 2.64	244 ± 2.59^a^	243 ± 2.51^a^	242 ± 2.49^a^
Final body weight (g)	308 ± 3.49	289 ± 3.58^a^	294 ± 3.31^b^	296 ± 3.38^c^
Body weight gain (g)	63 ± 0.85	45 ± 0.99^a^	51 ± 0.8^b^	54 ± 0.89^c^
Total food intake (g)	7,190 ± 3.49	7,145 ± 3.28^a^	7,175 ± 3.32^b^	7,180 ± 3.46^c^
Feed efficiency ratio	0.009 ± 0.24	0.006 ± 0.3^a^	0.007 ± 0.24^b^	0.008 ± 0.26^c^
Prostate weight (g)	0.35 ± 0.011	0.75 ± 0.018^a^	0.53 ± 0.009^b^	0.51 ± 0.01^c^
Prostate index %	0.11 ± 0.32	0.26 ± 0.5^a^	0.18 ± 0.27^b^	0.17 ± 0.29^c^

Values are presented as the mean ± SD (*n* = 6). As opposed to different letters in the same row reflecting a significant difference between treatments, the identical letters indicate no significant differences between treatments (*p* < 0.05). Feed efficiency = body weight gain/total food intake.

Benign prostatic hyperplasia is a non-cancerous and precancerous disorder of the prostate that is brought on by an overabundance of prostatic stromal and epithelial cells as a consequence of a discrepancy among both cell growth and death in the prostate (98). One of the most crucial indicators of the progress of BPH is an elevation in prostate weight, which coincides with a prostatic enlargement (99). The overstimulation of the prostate by sex hormones is the basis for animal models of BPH (100). In a model where testosterone is administered, this results in hyperplasia in the rat prostate, comparable to the morphological alterations in BPH in humans (101–103). We analyzed the prostate weight and index as the key indicators for the prostate hyperplasia treatment because, despite the treatment’s apparent influence on prostate weight rise, that should be associated with alterations in body weight. Comparatively to the normal control group, a significant rise in the prostate weight and the prostate index% was observed in the BPH group. Prostate weight and prostate index% were diminished in the treatment groups (1 and 2) containing soft cheese containing dehydrated primary and secondary DSP-liposomes compared to the BPH control group, suggesting that the dehydrated primary and secondary DSP-liposomes in the available soft cheese slowed the progression of BPH induced by testosterone.

#### 3.5.2 Biochemical parameters

Dihydrotestosterone (DHT) is widely regarded as the primary prostatic hormone that triggers the advancement and evolution of benign prostatic hyperplasia (BPH) (104). Internal male reproductive organ proliferation is significantly influenced by the steroid hormones testosterone and DHT, which have been correlated to BPH (28). The 5-reductase enzyme is accountable for converting testosterone to DHT, which performs a vital impact in BPH etiology and prostate progression. Comparing the BPH group to the normal control group, there was a significant diminishing in testosterone, DHT, and caspase-3. In contrast, compared to the BPH group, the testosterone and DHT levels were significantly reduced in groups 1 and 2, which were administered functional soft cheese containing dehydrated primary and secondary DSP-liposomes (105, 106). Furthermore, the study confirmed that the decline in caspase-3 levels attributed to testosterone had been counteracted by adding dehydrated primary and secondary DSP-liposomes, as evidenced by groups 1 and 2 compared to the BPH group. Consequently, our findings highlight that dehydrated primary and secondary DSP-liposomes are potent anti-proliferative agents that may be effectively used as prostatic hyperplasia therapy.

Benign prostatic hyperplasia may result from inflammation (107). Approximately 40% of those diagnosed with benign prostatic hyperplasia (BPH) display evidence of chronic inflammation, characterized by the presence of activated T cells, mast cells, and macrophages within the affected tissue (108). The study’s findings indicate that prostatic enlargement in rats may be attributed, at least in part, to pro-inflammatory cytokines such as TNF, IL-6, IL-10, and IL-1. According to the data presented in Table 4, TNF-, IL-6, IL-10, and IL-1 were more elevated in the BPH group than in the normal control group. The levels of TNF-, IL-6, IL-10, and IL-1 were reduced in the treatment groups (1 and 2) compared to the BPH group, suggesting that the high antioxidant content present in the DSP-liposomes acted as the catalyst for suppressing the responses of the inflammatory cytokines and inhibiting the anti-inflammatory IL-10 production.

**Table 4:** Effects of soft cheese containing dehydrated primary and secondary DSP-liposomes on hormones and cytokines.

Groups	Normal control	BPH control	Treatment group (1)	Treatment group (2)
Parameters				
Testosterone (ng/mL)	0.35 ± 0.18	1.35 ± 0.54^a^	0.95 ± 0.42^b^	0.99 ± 0.39^c^
Dihydrotestosterone (DHT) (pg/mL protein)	118.3 ± 1.05	235.4 ± 2.11^a^	173.4 ± 1.75^b^	168.2 ± 1.64^c^
Caspase-3 (ng/mg protein)	4.33 ± 1.03	1.95 ± 0.69^a^	2.94 ± 0.72^b^	3.09 ± 0.84^c^
TNF-α (pg/mL)	59.34 ± 1.37	85.44 ± 1.51^a^	66.32 ± 1.17^b^	64.29 ± 1.21^c^
IL-6 (pg/mL)	37.28 ± 1.67	65.31 ± 1.99^a^	44.23 ± 1.46^b^	42.72 ± 1.19^c^
IL-10 (pg/mL)	51.34 ± 2.47	79.65 ± 2.98^a^	61.28 ± 2.08^b^	58.72 ± 2.31^c^
IL-1β (pg/mg protein)	87.39 ± 1.98	139.26 ± 2.11^a^	109.24 ± 1.65^b^	101.78 ± 1.17^c^

Values are presented as the mean ± SD (*n* = 6). As opposed to different letters in the same row reflecting a significant difference between treatments, the identical letters indicate no significant differences between treatments (*p* < 0.05).

The role of inflammation has been identified as significant in developing benign prostatic hyperplasia (BPH) induced by testosterone (109). The antioxidant enzymes, namely CAT, GSH, GPx, GR, and SOD, typically safeguard prostatic cells by stimulating the body’s defense mechanism against oxidative stress-induced injury (110). Antioxidant enzymes perform a crucial impact in mitigating the harmful effects of free radicals by catalyzing their conversion into relatively benign oxygen species (111). The rate of membrane lipid peroxidation is influenced by a multitude of variables, including the strength of the antioxidant defenses in the surrounding environment, cell membrane lipid structure, and the presence of Cd-induced radicals that are free and excited-state molecules that can initiate propagation. The BPH group in the current study noticed the oxidative stress and failure of the antioxidant mechanism in it compared to the normal control group, as evidenced by our results in Table 5, where MDA was significantly elevated, and CAT, GSH, GPx, GR, and SOD were significantly reduced. Testosterone administration led to an elevated level of oxidative stress and an unnatural sway in the prostatic tissues’ proliferation and apoptosis rates (112, 113). It has been demonstrated that oxidative stress plays a part in BPH (114). Testicular GSH depletion was identified in the present study as one factor causing the rise in oxidative damage to lipids and proteins. The observed substrate glutathione (GSH) deficiency explains glutathione peroxidase’s (GPx) diminished performance. Direct antioxidant action performed by glutathione in response to singlet oxygen, peroxy radicals, and superoxide radicals leads to oxidized glutathione (GSSG) along with other disulfides, which substantially impact regulating a wide variety of cellular activities.

**Table 5:** Effects of soft cheese containing dehydrated primary and secondary DSP-liposomes on oxidative stress markers.

Groups	Normal control	BPH control	Treatment group (1)	Treatment group (2)
Parameters				
MDA (nmol/mg protein)	1.44 ± 0.32	3.63 ± 0.92^a^	1.61 ± 0.56^b^	1.55 ± 0.43^c^
GSH (nmol/mg protein)	2.47 ± 0.86	1.09 ± 0.24^a^	1.89 ± 0.53^b^	1.97 ± 0.59^c^
SOD (nmol/mg protein)	61.22 ± 2.13	35.29 ± 1.62^a^	51.08 ± 1.93^b^	52.28 ± 1.19^c^
CAT (U/mg protein)	0.98 ± 0.05	0.58 ± 0.06^a^	0.79 ± 0.08^b^	0.81 ± 0.08^c^
GPx (μg/mg protein)	102.67 ± 3.54	71.26 ± 2.95^a^	87.12 ± 1.99^b^	89.32 ± 2.04^c^
GR (nmol/mg protein)	95.16 ± 1.68	69.33 ± 2.11^a^	85.29 ± 2.31^b^	86.89 ± 2.46^c^

Values presented as the mean ± SD (*n* = 6). As opposed to different letters in the same raw reflecting a significant difference between treatments, the identical letters indicate no significant differences between treatments (*p* < 0.05).

The study demonstrated that dehydrated primary and secondary DSP-liposomes administered to the treatment groups (1 and 2) effectively suppressed the elevated levels of lipid peroxidation products, depletion of both GSH and CAT compared to the BPH group. Conversely, dehydrated primary and secondary DSP-liposomes mitigated oxidative damage in prostate tissues. The data mentioned above are consistent with the antioxidant qualities of DSP-liposomes (115). Phenolic and flavonoid compounds of DSP-liposomes had anti-inflammatory and antioxidant actions that allow them to stabilize reactive oxygen radicals and perform antioxidation (116). The findings indicate that DSP-liposomes can impede the advancement of BPH, thereby holding promise as a potential therapeutic intervention for BPH.

The findings reported in Table 6 revealed that, in contrast to the normal control group, the BPH group exhibited significantly elevated levels of total cholesterol, LDL, and triglycerides and significantly reduced levels of HDL. Conversely, the treatment groups (1 and 2) that administered soft cheese containing primary and secondary DSP-liposomes exhibited a noteworthy diminishing in total cholesterol, LDL, and triglycerides, as well as a substantial rise in HDL, in contrast with the BPH group (117, 118). The data analysis revealed no statistically significant difference in the levels of creatinine and urea in the BPH group in contrast with the normal control group and among all of the treatment groups (1 and 2) when compared to the BPH group. The study noticed an enhancement in the performance of the liver, as evidenced by a noteworthy reduction in hepatic enzyme activities in the treatment groups (1 and 2) that were administered soft cheese containing primary and secondary DSP-liposomes. This outcome is attributed to the existence of phenolic and flavonoid compounds in DSP-liposomes when compared to the BPH group in comparison to the opposing group, the BPH group exhibited a noticeable rise in AST, ALT, and ALP, indicating a substantial alteration in the liver’s functionality resulting from testosterone induction (44, 47).

**Table 6:** Effect of soft cheese containing dehydrated primary and secondary DSP-liposomes on lipid profiles, hepatic function biomarkers, and kidney function biomarkers.

Groups	Normal control	BPH control	Treatment group (1)	Treatment group (2)
Parameters				
Total cholesterol (mg/dL)	94.1 ± 4.67	86.8 ± 5.34^a^	89.2 ± 4.29^b^	89.8 ± 4.32^c^
HDL (mg/dL)	53.7 ± 3.76	42.9 ± 3.52^a^	49.4 ± 3.15^b^	49.9 ± 3.25^c^
LDL (mg/dL)	32.8 ± 2.58	40.5 ± 3.02^a^	31.3 ± 2.64^b^	31.8 ± 3.36^c^
Triglycerides (mg/dL)	48.9 ± 2.42	63.4 ± 3.59^a^	44.8 ± 3.31^b^	45.2 ± 3.35^c^
Creatinine (mg/ dL)	0.96 ± 0.05	0.94 ± 0.03^a^	0.93 ± 0.04^a^	0.93 ± 0.04^a^
Urea (mg/dL)	34.5 ± 1.24	35.2 ± 1.31^a^	33.6 ± 1.19^a^	33.9 ± 1.26^a^
AST (U/L)	127.8 ± 2.16	152.7 ± 3.04^a^	109.3 ± 2.37^b^	110.6 ± 2.41^c^
ALT (U/L)	95.4 ± 1.16	175.6 ± 2.02^a^	119.2 ± 1.45^b^	113.8 ± 1.39^c^
ALP (U/L)	78.3 ± 3.01	66.4 ± 3.68^a^	70.4 ± 2.72^b^	71.7 ± 2.82^c^

Values presented as the mean ± SD (*n* = 6). As opposed to different letters in the same raw reflecting a significant difference between treatments, the identical letters indicate no significant differences between treatments (*p* < 0.05).

Based on our biological findings, it was observed that primary and secondary DSP-liposomes yielded similar outcomes. However, the dehydrated secondary DSP-liposomes exhibited superior results due to using a chitosan-protected liposome technique, which prevented aggregation by encapsulating it with maltodextrin until the bioactive components were released in the stomach. This viable technique may be recommended for employment in managing benign prostatic hyperplasia (BPH).

## 4 Conclusion

Liposomal systems for encapsulating phenolics are considered a viable strategy for protecting them against adverse environmental factors and improving their bioaccessibility. The findings of this investigation indicate that applying a chitosan layer onto liposomes enhanced the liposomal system structural integrity. On the other hand, the encapsulation of date seed phenolics (DSP) within liposomes was accomplished effectively and the liposomes were successfully integrated into a soft cheese product. This technique appears to be a viable option for enhancing the nutritional value of exceptionally soft cheese. Furthermore, using dehydrated secondary DSP-liposomes has a favorable prophylactic impact on benign prostatic hyperplasia progression. Additional empirical investigations may be necessary to validate the current results before assessing their importance for the treatment of benign prostatic hyperplasia (BPH).

## Data availability statement

The raw data supporting the conclusions of this article will be made available by the authors, without undue reservation.

## Ethics statement

The animal studies were approved by Medical Research Ethical Committee approved (Approval No. 7411062021) under the United Kingdom’s Animals (Scientific Procedures) Act, 1986, and associated guidelines, EU Directive 2010/63/EU for animal experiments (Publication No. 85–23, revised 1985). The studies were conducted in accordance with the local legislation and institutional requirements. Written informed consent was obtained from the owners for the participation of their animals in this study.

## Author contributions

DM: Conceptualization, Formal analysis, Methodology, Software, Validation, Writing – original draft. TE: Conceptualization, Formal analysis, Investigation, Methodology, Software, Visualization, Writing – original draft. DB: Writing – original draft, Writing – review & editing. MH: Data curation, Methodology, Validation, Writing – review & editing. MB: Investigation, Software, Writing – original draft. ME: Conceptualization, Investigation, Methodology, Software, Validation, Visualization, Writing – original draft, Writing – review & editing.
